# Experience with oral mexiletine in primary erythromelalgia in children

**DOI:** 10.4103/0256-4947.55316

**Published:** 2009

**Authors:** Javeed Iqbal, Mushtaq I. Bhat, Bashir A. Charoo, Wajid A. Syed, Mushtaq A. Sheikh, Imtiyaz N. Bhat

**Affiliations:** From the Department of Pediatrics and Neonatology, Sher-i-Kashmir Institute of Medical Sciences, Srinagar, Jammu and Kashmir, India

## Abstract

Primary erythromelalgia is characterized by burning pain, redness, and warmth in the extremities. We present two cases of primary erythromelalgia both of whom presented with a history of several months of severe burning pain in both hands and feet. Both patients had received multiple pain medications with no improvement in symptoms. Pain was relieved by putting affected parts in ice cold water, which resulted in immersion injury of the affected parts. Both patients stopped taking part in school and social activities. We tried oral mexiletine, a class Ib antiarrythmic agent, in view of its reported role in various chronic painful conditions. Dramatic improvement was observed with its use. Both patients improved after several weeks of use, and there were fewer soaking episodes. We observed no adverse effects with mexilitine therapy.

Erythromelalgia is a disorder characterized by severe burning pain, mainly involving the extremities and associated with erythema and increased temperature of the affected parts. Warmth aggravates the symptoms and cold provides relief.^1^ Frequent immersion into ice cold water for relief of pain is a constant feature of the disease and is considered pathognomonic.^2^ The pain associated with erythromelalgia is often resistant to treatment. Although a wide variety of therapies have been tried, no effective treatment is yet available. In a review of data for 168 patients with erythromelalgia at the Mayo clinic it was found that patients had used 84 different types of medications.^3^ We present two patients with primary erythromelalgia, both of whom had received a number of painkillers before reporting to us. Both patients were given a trial of oral mexilitene and showed remarkable response.

## CASE 1

**Figure 1:**
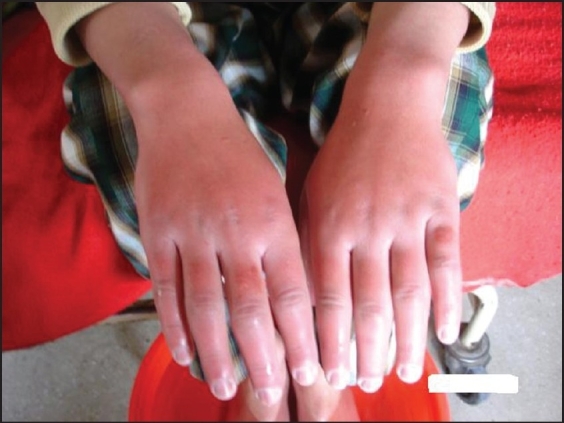
Photograph of a 12-year-old child showing erythromelalgia of both hands.

A 12-year-old male presented to us with a history of severe burning pain in both hands and feet associated with redness and erythema for the previous 3 months. Pain was aggravated by heat and classically relieved by placing the hands and feet in ice cold water. Because of the severe pain, the patient would keep both hands and feet immersed in ice cold water most of the time, as a result of which the patient developed a severe immersion injury (Figure 1). There was no prior history of drug intake or immunization.

Clinical examination revealed a weight of 35 kg. There was no abnormality apart from the immersion injury and no neurological deficit. The complete blood count was normal apart from a hematocrit of 45%. RBC mass was 34 mL/kg. A complete metabolic workup, liver function tests, a work-up for connective tissue disorders (antinuclear antibodies, anti-double stranded DNA), coagulation tests, nerve conduction velocity (NCV), electromyography (EMG) were within normal limits.

Initially, the patient was given aspirin (15 mg/kg/day) for about 1 week with no improvement in symptoms. Subsequently, morphine (2.5 mg IV every 4 hours) was added, but again the patient showed no improvement. Treatment was discontinued after 2 weeks. The patient was started on oral mexiletine initially at 100 mg three times a day, which was increased subsequently to 200 mg three times a day. No adverse effect was observed. Improvement began after 2 weeks of therapy. The frequency, duration, and intensity of the pain episodes continued to decrease significantly and by 5 weeks of therapy all symptoms completely resolved. As of 6 months from discharge the patient had experienced very few episodes of mild burning pain, which did not incapacitate the patient. The patient reported no side effects from mexiletine therapy.

## CASE 2

An 8-year-old male child presented with burning pain in both hands and feet associated with erythema for the previous month. Classically, the pain was relieved by immersing the affected part in cold water. Symptoms were aggravated by heat. The patient had developed immersion injury to both hands. There was no significant illnesses in the past prior to the disease. The patient was a product of a nonconsanguinous marriage with no family history of any myeloproliferative or connective tissue disorder. Clinical examination revealed a weight of 25 kg, and immersion injury of the left foot. A complete blood count revealed a hematocrit of 36%. A complete metabolic workup, liver function tests, workup for connective tissue disorders (antinuclear antibodies, anti-double stranded DNA), coagulation tests, nerve conduction velocity (NCV), electromyography (EMG) were within normal limits. Initially, the patient was treated with aspirin for about 1 week with no improvement. Subsequently, gabapentin was added. He showed a partial response to combination therapy, but the patient was still not able to participate in school and other social activities. He was started on oral mexiletine, initially 100 mg three times a day, which was increased subsequently to 200 mg three times a day. The frequency, duration, and intensity of the pain episodes continued to decrease significantly. During regular follow-up for two months after discharge the patient experienced no side effects from mexilitine and had marked improvement in symptoms with only a few episodes of pain, which were not incapacitating.

## DISCUSSION

Because of lack of understanding of the pathophysiology and its low prevalence, treatment of erythromelalgia is poorly understood and often difficult to treat. Early studies suggested that aspirin effectively relieved symptoms, but aspirin appears to be significantly more effective when there is an associated thrombocythaemia and polycythemia vera.^2^ Various pharmacologic trials have been conducted with varied results. These include use of tricyclic antidepressants, gabapentin, anticonvulsants, and others.^4^,^5^ Mexiletine has been used in various chronic painful conditions and has been found quite effective.^6^ It is a class IB orally active anti-arrhythmic agent, a sodium-channel inhibitor that shortens the action potential duration. Mutations in regions of chromosome 2q, which contain a cluster of sodium channel genes, have been identified among patients with primary erythromelalgia.^7^ Sodium-channel blockers like mexiletine are widely used to treat neuropathic pain. Subacute or chronic inflammation also produces profound changes in the excitability of primary afferent neurons innervating the inflamed tissue.^8^ Recent evidence suggests that post-translational modifications or abnormal expression of sodium channels in dorsal root ganglion neurons occurs after tissue inflammation.^9^ In fact, there are few published case reports on the benificial effect of oral mexiletine in primary erythromelalgia.^10^ Nathan et al^11^ reported on an 11-year-old, white male child with primary erythromelalgia who demonstrated marked improvement following treatment with a combination of intravenous lidocaine and oral mexiletine therapy. Kuhnert et al^10^ also demonstrated a similar effect in an adult patient with severe erythromelalgia. Mexiletine has also been found useful in inflammatory pain in experimental animals^12^ and in the pain associated with diabetic neuropathy.^13^ Mexiletine provides analgesia by blocking sodium channels at both peripheral and central sites. The ability of mexiletine to selectively block nerves in a more depolarized state makes this drug useful for blocking pain pathways in which neural activity is increased.

In summary, we present two cases of primary erythromelalgia who were suffering from a severe burning pain with a severe degree of immersion injury whose symptoms were signficantly controlled by oral mexiletine with no recognizable side effect of therapy.
